# Factors associated with self-rated health among North Korean defectors residing in South Korea

**DOI:** 10.1186/1471-2458-14-999

**Published:** 2014-09-26

**Authors:** Bo-Ram Wang, Shieun Yu, Jin-Won Noh, Young Dae Kwon

**Affiliations:** Catholic Institute for Healthcare Management, the Catholic University of Korea, 222 Banpo-daero, Seocho-gu, Seoul, 137-701 Korea; Department of North Korean Studies, Korea University, 2511 Sejong-ro, Sejong, 339-700 Korea; Department of Healthcare Management, Eulji University, 212 Yangji-dong, Sujeong-gu, Seongnam, Gyeonggi-do 461-713 Korea; Department of Humanities and Social Medicine, College of Medicine and Catholic Institute for Healthcare Management, The Catholic University of Korea, 222 Banpo-daero, Seocho-gu, Seoul, 137-701 Korea

## Abstract

**Background:**

The number of North Korean refugees entering South Korea has increased recently. The health status of refugees is a significant factor in determining their success in resettlement; therefore, this study examined both the self-rated health status of North Korean defectors who have settled in South Korea and the factors associated with their self-rated health status.

**Methods:**

This study utilized data gained from face-to-face interviews with 500 North Korean defectors who arrived in South Korea in 2007. The interviews were structured and conducted by ‘Yonsei University Research Team for North Korean defectors’. A stepwise multivariable linear regression was performed to determine the factors associated with their self-rated health status.

**Results:**

North Korean defectors who were female, elderly, or had low annual household income, disability or chronic diseases reported lower health status. However, self-rated health status was higher among those who had settled in South Korea for 18 months or more, who were satisfied with government support or their current life, and who had experienced more traumatic events in North Korea.

**Conclusions:**

Government policies and refugee assistance programs should consider and reflect the factors relevant to the health status of North Korean defectors.

## Background

Since the 1945 intervention of the United States and the Soviet Union in the Korean War, the ideologically disparate governments of South and North Korea have remained mutually hostile. The number of North Korean refugees has increased since the mid-1990s due to the worsening economic crisis, food shortages caused by natural disasters, and poor living conditions in North Korea [1]. The number of North Koreans fleeing to South Korea increased from 1,400 in 2000 to 10,000 in 2006, reaching 23,000 in 2012 [2, 3].

Post-arrival health assessment has revealed that many North Korean defectors suffer from various physical/mental illnesses and that diseases they are suffering from remain dormant due to stress until they arrive in South Korea [4]. It is assumed that they had experienced poor living conditions in North Korea, as well as numerous life-threatening events during their illegal stay in a tertiary country, such as China and Russia, before entering South Korea [1, 4]. Moreover, the stress from socio-cultural and economic assimilation in South Korean society increases the risk of psychological and physical disorders [4–6]. These health vulnerabilities often result in passivity among North Korean defectors with regards to finding employment and participating in government social adjustment programs [4, 7, 8].

The health status of refugees is one of many significant factors that determine the success of their resettlement [9]. Previous studies have shown that newly arrived refugees frequently require immediate care for physical and psychological traumas and illnesses [10–12] because they are exposed to various forms of harm during migration [13]. Furthermore, a high level of stress due to assimilation into a new society may increase the risk of developing chronic diseases [10, 13]. For these reasons, host countries examine the health of refugees upon arrival and provide a variety of medical support services [14–18]. The South Korean government has given North Korean defectors refugee status and provides them with medical services under the Medical Aid Act [19]. In 2004, Hanawon, the government resettlement center for North Korean refugees that provides a variety of services like education and mentorship programs, also founded the Hana Clinic to improve the health and well-being of refugees [4].

The health status of North Korean defectors is a significant indicator of their success in settling into South Korean society. Several studies have investigated North Korean refugee health in South Korea; however, most have focused solely on mental health related to trauma exposure [1, 20, 21]. A limited number of studies have investigated the physical health of refugees. Also these studies were restricted in scope because they only used a univariable analysis [4] or lacked follow-up health assessments after initial data was obtained upon their arrival thereby precluding any assumptions regarding their health conditions during the assimilation process [22, 23]. This study investigated the self-rated health status of North Korean defectors who have lived in South Korea for a specific period of time, and it identified the factors related to their self-rated health status.

## Methods

### Subjects and data

From a total of 2,138 North Korean defectors aged 20–69 years, who had all entered South Korea between January and December 2007, 500 individuals agreed to participate in this cross sectional study. Of the original total, we were unable to contact 1,447 individuals, and 107 individuals refused to participate. Another 84 individuals either abandoned the study or were unavailable for face-to-face interviews. There were difficulties with the application of random sampling due to an insufficient number of participants; the situation was exacerbated by the fact that North Korean defectors are afraid of revealing their true identities. A total of 500 individuals participated in interviews from April 6th to May 25th of 2009; however, two participants were excluded after the interviews had taken place due to insufficient data, resulting in a total of 498 participants taking part in this study. The interviews consisted of quantitative and qualitative observation in accordance with qualitative research review guidelines - RATS [24]. The study protocol and required consent forms were verified and approved by the Institutional Review Board of the Severance Hospital before the interviews took place.

### Variables

This study considered the general health status of each subject, measured by a self-reported health measurement scale, as the dependent variable. Self-rated health closely corresponds to a person’s objective physical health status [25] and plays an important role in influencing health-seeking behavior, including personal health practices and the use of healthcare services. Self-rated health was assessed using a five-point scale: excellent (5), very good (4), good (3), fair (2), poor (1).

Independent variables included ‘demographic and socio-economic factors’, ‘special characteristics for defectors’, and ‘health-related factors’. Demographic and socio-economic variables included gender, age, marital status, size of household, education level, religion, employment status, annual household income, and type of health insurance. The values of all variables were taken from data analyzed on December 31, 2008; education level was assessed based on the level of education completed in North Korea. Marital status was classified as single, married, or unmarried, and the unmarried category was further divided into divorced, separated or widowed groups. Household size was classified as single or multiple. The level of education completed in North Korea was categorized as elementary, secondary, or some college or higher. Annual income included earned income and government aid. Natural logarithm was used to compensate for skewed distribution and variation of household income across study participants. Type of health insurance was categorized as Medical Aid or National Health Insurance (NHI).

Variables representing special characteristics of the North Korean defectors included duration of exile, duration of residence in South Korea, level of satisfaction with support from the South Korean government, satisfaction with current life, traumatic experiences in North Korea, and traumatic experiences during escape. The duration of exile was defined as the period between the date of escape until December 31, 2008, which includes time spent in other countries and South Korea; it was classified as less than 5 years, 5–10 years, or 10 years or longer. Duration of residence in South Korea was defined as the period between an individual’s arrival date until December 31, 2008, and it was categorized as less than 18 months or 18 months or longer. The level of satisfaction with government support and current life satisfaction were assessed using a five-point scale, with a higher score indicating greater satisfaction. Traumatic experiences were measured using questionnaires developed by Kang [26], which include 17 questionnaires measuring traumatic experiences in North Korea and 18 questionnaires assessing traumatic experiences during travel, with one point allocated for each question [27]. The Cronbach’s alpha values were 0.750 for traumatic experiences in North Korea and 0.761 for traumatic experiences during escape, and thus the coefficient proved the reliability of two variables. Health-related variables include disability status, chronic diseases, and drinking habits. Drinking habits were categorized as no drinking, one or fewer drinks a month, or two or more drinks a month.

### Statistical analysis

A t-test and a one-way ANOVA analysis were performed to investigate the socio-economic and demographic characteristics of study participants, the special characteristics of North Korean defectors, and the health-related characteristics; differences in self-rated health were also identified. A simple regression analysis was performed for continuous independent variables. Because the distribution of self-rated health resembled the shape of a normal distribution, a stepwise linear regression was applied for the analysis of independent relations in the multivariable model [28]. Data was analyzed using the Statistical Package for the Social Sciences, version 18.0 (SPSS Inc., Chicago, IL, USA).

## Results

### Self-rated health according to the general characteristics of study participants

Table 1 presents self-rated health according to participant characteristics. 13.1% of participants rated their health as excellent; 32.3% as very good; 25.3%, as good; 22.5%, as fair; and 6.8%, as poor. The mean value of self-rated health score for study participants was 2.78. The self-rated health score for males (3.10) was significantly higher than that of females (2.70) (*P* = 0.001) and declined with age (*P* < 0.001). The significantly highest score (3.05) was found in subjects who were single, while the lowest (2.52) was found in those who were divorced, separated, or widowed (*P* < 0.001). Participants who only had an elementary education had the significantly highest score of 3.34, whereas individuals who completed secondary school had the lowest score of 2.69 (*P* = 0.003). Study participants with a religious affiliation scored significantly lower (2.63) on their subjective health score than those with no such affiliation (2.91) (*P* = 0.006). Employed participants scored significantly higher (2.98) than the unemployed (2.57) (*P* < 0.001), and the score increased with a growth in annual household income (*P* < 0.001). Self-rated health of individuals in the Medical Aid beneficiary group (2.63) was significantly lower than those with NHI (3.27) (*P* < 0.001). The rating was significantly high among participants who had been living outside of North Korea for 10 years or longer (3.03) (*P* = 0.035) and among those who had been residing outside of North Korea for 18 months or longer (2.89) (*P* = 0.029). Individuals who reported significantly high levels of satisfaction with government support (*P* < 0.001) and their current life (*P* < 0.001) also had a higher self-rated health score. Subjective health status significantly increased among those who had traumatic experiences either in North Korea (*P* < 0.001) or during escape (*P* = 0.020). Individuals with disabilities (1.94) or chronic diseases (2.00) had significantly lower scores than those without disabilities (2.84) (*P* < 0.001) or chronic diseases (3.36) (*P* < 0.001). Individuals who consumed no alcohol (2.60) had a significantly lower self-rated health score than those who drink alcohol (2.94 and 2.78 for those who drink one or two or more drinks per month, respectively) (*P* = 0.008).

**Table 1:** **Relationships between general characteristics and self-rated health in North Korean defectors**

	N (%)	Mean ± SD	Beta	t/F	***P***-value
Self-rated health		2.78 ±1.14			
Gender					
Male	101 (20.3)	3.10 ± 1.17		3.21	.001
Female	397 (79.7)	2.70 ± 1.12			
Age (years)		35.8 ± 8.3	-.306	-7.15	<.001
Marital status					
Single	152 (30.5)	3.05 ± 1.16		8.43	<.001
Married	199 (40.0)	2.75 ± 1.08			
Divorced/separated/widowed	146 (29.3)	2.52 ± 1.15			
Size of household					
1	190 (38.2)	2.83 ± 1.16		.66	.512
≥2	276 (55.4)	2.76 ± 1.13			
Education in NK					
Elementary school graduate	38 (7.6)	3.34 ± 1.17		6.04	.003
Secondary school graduate	337 (67.7)	2.69 ± 1.13			
Some college or higher	123 (24.7)	2.85 ± 1.12			
Religion					
Yes	237 (47.6)	2.63 ± 1.20		2.78	.006
No	261 (52.4)	2.91 ± 1.07			
Job					
Yes	252 (50.6)	2.98 ± 1.09		-3.98	<.001
No	220 (44.2)	2.57 ± 1.16			
^*^ln (annual household income)		16.46 ± .74	.243	4.75	<.001
Health insurance type					
Medical Aid	370 (74.3)	2.63 ± 1.13		-5.47	<.001
NHI	120 (24.1)	3.27 ± 1.03			
Exile duration					
<5 years	256 (51.4)	2.73 ± 1.15		3.37	.035
5≤ ~ <10 years	135 (27.1)	2.67 ± 1.07			
≥10 years	104 (20.9)	3.03 ± 1.17			
Residence period in SK					
<18 months	243 (48.8)	2.66 ± 1.13		-2.19	.029
≥18 months	253 (50.8)	2.89 ± 1.14			
Satisfaction with SK government support	3.78 ± 1.00	.159	3.58	<.001	
Life satisfaction	3.22 ± .89	.183	4.15	<.001	
Traumatic experiences in NK	11.93 ± 3.18	1.171	3.86	<.001	
Traumatic experiences during escape	15.05 ± 2.73	.104	2.33	.020	
Disability					
Yes	36 ( 7.2)	1.94 ± 1.04		4.65	<.001
No	462 (92.8)	2.84 ± 1.12			
Chronic disease					
Yes	214 (43.0)	2.00 ± .89		16.4	<.001
No	284 (57.0)	3.36 ± .95			
Drinking					
None	215 (43.2)	2.60 ± 1.12		4.94	.008
Once a month or less	127 (25.5)	2.94 ± 1.15			
Twice a month or more	156 (31.3)	2.78 ± 1.13			
Total	498 (100.0)				

*Natural logarithmic transformation was applied to annual household income (amount of Korean won).

NK, North Korea; SK, South Korea; NHI, National Health Insurance.

### Factors associated with self-rated health

Table 2 presents the result of the stepwise multivariable regression analysis, which sought to identify factors influencing self-rated health. The regression model is statistically significant (F = 32.64, *P* < 0.001) and the variables explain 42.1% of variance in self-rated health (adjusted R^2^ = 0.421). The statistically significant variables were gender (*P* < 0.001), age (*P* < 0.001), annual household income (*P* = 0.017), duration of residence in South Korea (*P* = 0.021), satisfaction with government support (*P* = 0.037), life satisfaction (*P* = 0.001), traumatic experiences in North Korea (*P* = 0.039), and chronic diseases (*P* < 0.001). The self-rated health score among female respondents was 15.4% lower than that of males (*P* < 0.001). The score decreased with age (*P* < 0.001) but increased with growth in annual household income. Respondents who had been living in South Korea for 18 months or longer had a score that was 9.6% higher than those who had been living there for fewer than 18 months (*P* = 0.021). The self-rated health score increased among those with higher satisfaction with government support (*P* = 0.037) and their current life (*P* = 0.001), and among those with high scores in their evaluation of traumatic experiences in North Korea (*P* = 0.039). Individuals with chronic diseases had a 4.6% lower self-rated health score than those without chronic diseases (*P* < 0.001).

**Table 2:** **Multivariable linear regression analysis for self-rated health in North Korean defectors**

	Beta	***P***-value	VIF
Gender (ref. male)			
Female	-.154	<.001	1.111
Age (years)	-.184	<.001	1.167
Marital status (ref. single)			
Married	-.043	.303	1.025
Divorced/separated/widowed	.007	.874	1.222
Education in NK (ref. elementary school graduate)			
Secondary school graduate	-.008	.843	1.049
Some college or higher	.017	.687	1.064
Religion (ref. no)			
Yes	-.056	.181	1.036
Job (ref. no)			
Yes	.048	.307	1.300
^*^ln (annual household income)	.102	.017	1.095
Health insurance type (ref. Medical Aid)			
National Health Insurance	.057	.184	1.119
Exile duration (ref. <5 years)			
5≤ ~ <10 years	-.001	.985	1.038
≥10 years	.053	.211	1.098
Residence period in SK (ref. <18 months)			
≥18 months	.096	.021	1.019
Satisfaction with SK government support	.094	.037	1.204
Life satisfaction	.141	.001	1.166
Traumatic experience in NK	.089	.039	1.112
Traumatic experiences during escape	-.006	.901	1.308
Disability (ref. no)			
Yes	-.074	.077	1.041
Chronic disease (ref.no)			
Yes	-.460	<.001	1.171
Drinking (ref. not drinking)			
Once a month or less	.066	.117	1.067
Twice a month or more	.035	.445	1.223

F-value = 32.64 (*P* <.001).

R^2^ (adjusted R^2^) = .434 (.421).

Number of observations = 498.

*Natural logarithmic transformation was applied to annual household income (amount of Korean won).

ref. reference; NK, North Korea; SK, South Korea; KRW, Korean won; VIF, variance inflation factor.

## Discussion

This study evaluated the self-rated health of 498 North Korean defectors who entered South Korea in 2007; it also analyzed the factors associated with their self-rated health. This study assessed their overall health conditions due to the difficulty involved in determining the appropriate objective health status measurements that would provide a comprehensive picture of their physical and mental health status. The mean value of self-rated health status score among these refugees was relatively low: 2.78 out of 5. This result is consistent with previous findings that reported 11.8-28.4% of North Korean refugees who considered their health condition to be good, as opposed to 40.8% of South Koreans; this shows a significantly lower level of self-rated health among North Korean refugees [4, 29, 30].

With regards to the demographic characteristics of participants, female and elderly respondents tended to have a lower health status, which is a trend observed not only among refugees but also in the general population [31, 32]. It is well-known that individuals with a higher level of education tend to have better health status and longer life expectancy [32–34]. It has been reported that a lower level of education results in income inequality, particularly among males, which in turn results in a lower health status [35]. However, in this study, the level of education received in North Korea had no association with the self-rated health score. It is assumed that diplomas obtained in North Korea have no relationship with resettlement in South Korea. The socio-economic status of most North Korean defectors declines in South Korean society, and many of them do not have any opportunities to use their previously-acquired abilities and experiences [8].

Individuals with a lower annual household income had lower self-rated health scores. A previous study showed that a refugee’s perceived economic hardship is related to greater decrease in physical health status [9]. In 2008, the average annual family income among North Korean defectors was 8,780,000 Korean won (KRW), which is significantly lower than the income of South Koreans (30,790,000 KRW) surveyed between 2007 and 2009 [36]. Only a small portion of North Korean refugees have stable employment, while most are unemployed or are engaged in entry-level work [8]. According to a survey conducted in 2001, the income of North Korean defectors was only 57% of the average income of South Koreans [8], which could be explained by unstable employment and the unemployment crisis in South Korea [37]. The relationship between individual economic status and health is not limited to refugees; South Koreans with low socioeconomic status are also likely to have low self-rated health [33]. Most North Korean refugees belong to the low socio-economic stratum in South Korea, and so their household income level is likely associated with their health status.

Higher levels of satisfaction with government support, as well as greater life satisfaction, were associated with an increase in self-rated health. This may be explained by the fact that government assistance is linked to the economic status of refugees in South Korea, and therefore it may also be related to life satisfaction. The South Korean government provides various types of support for North Korean refugees, spanning from their arrival until gaining stable residence, in order to provide the necessary conditions for initial assimilation [37]. The South Korean government has also designated refugees as Medical Aid beneficiaries in order to provide them with basic medical health care services [4]. It is most likely that defectors who actively utilize and are satisfied with this government support have higher self-rated health. Considering that self-rated health status of defectors increased with higher satisfaction with government supports, it is necessary to seek a way of improving satisfaction with government support programs. Although the South Korean government provides Medical Aid to refugees to ensure access to basic medical services, this access is limited due to high out-of-pocket costs for those services not covered by NHI. All Medical Aid beneficiaries in South Korea are economically vulnerable and therefore face difficulties paying for medical services outside NHI coverage [38]. The economic status of North Korean defectors is particularly low; therefore, their lack of access to necessary health care services may be more serious. Although the insurance type was not a significant factor associated with self-rated health in the multivariable model, participants receiving Medical Aid reported lower self-rated health. Therefore, the development of alternatives may assist in increasing refugee access to health care services. Further research is necessary in order to identify associations between health insurance type and medical utilization of defectors. Individuals with longer length of stay (>18 months) in South Korea tended to have better self-rated health. This result may be indicative of the fact that North Korean defectors escaped from poor living conditions, such as malnutrition [1] and have since been able to access better health care services [9], thereby increasing self-rated health. Another interpretation suggests improvement in emotional and physical stability after resettlement in South Korea; a previous study reported that immigrants and refugees with longer residency in a new country experience greater assimilation into a new society and culture [39]. However, several studies have reported that a longer length of stay in the United States is actually associated with an increased prevalence of obesity and cardiovascular diseases in immigrants due to the adoption of unhealthy eating habits and lifestyle [40–42]. North Korean defectors with chronic diseases reported lower self-rated health; although this study was unable to identify the exact onset of such diseases, their prevalence does increase among refugees in the process of resettlement [4]. This result likely suggests that preventive care, early detection and intervention are essential during the early phase of resettlement for the prevention and control of chronic diseases.

The health status of refugees is a key factor affecting resettlement in host countries [9], and many countries provide refugees with various health care services upon arrival to improve their health status. The Australian government provides refugees with a Medicare program and additional services including dental and mental health care [16]. Other countries, such as Canada, United Kingdom, Netherlands, and the United States also provide comprehensive healthcare services to refugees upon arrival [14, 15, 17, 18]. It is recommended that the South Korean government provide newly-arrived refugees with prompt integrated treatment and continuous healthcare services to improve their health status, which is a key determinant of their success in resettlement.

North Korean defectors have undergone numerous traumatic experiences, both in North Korea and during their defection, which can hinder their successful settlement in South Korea [43] and this may be related to their self-rated health. The results from this study showed that higher levels of traumatic exposure in North Korea were associated with higher levels of self-rated health. This may be explained by posttraumatic growth and resilience. Posttraumatic growth is defined as the experience of positive changes resulting from a struggle with previous psychological trauma and stress [44]. Although the negative effects of traumatic experiences cannot completely disappear, individuals who experience posttraumatic growth may expand their understanding of life and develop new coping skills, resulting in personal growth and recovery from previous traumas [45–47]. Resilience also functions as a buffer against the negative effects of traumatic experiences that affect positive adaption [43]. Previous studies revealed that resilience has a positive effect on one’s physical and mental adaption caused by trauma or high levels of stress [48, 49]. North Korean defectors who experienced traumatic incidents may have undergone positive personal development and may have increased their resilience through struggles with previous trauma. It is assumed that individuals with a high degree of exposure to traumatic events tend to have better self-rated health once they overcome the trauma.

This study attempted to utilize random sampling, however, it was practically impossible since the total number of participants was very low; North Korean defectors tend to avoid identity exposure, and they are passive in face-to-face interviews. Despite attempts to include as many refugees as possible in the interviews, the results may not be generalizable to all North Korean defectors residing in South Korea. Also, this study analyzed data collected from a cross-sectional study, and so it has limitations in detecting causal relations between independent variables and self-rated health.

## Conclusions

This study analyzed the factors associated with self-rated health among North Korean defectors living in South Korea. The results showed that the length of stay in South Korea, satisfaction with South Korean government support, traumatic experiences in North Korea, and life satisfaction are significant factors in the self-rated health of defectors when other variables such as demographic, socio-economic, and health-related factors are controlled. These findings should be considered and reflected in government policies and refugee support programs in order to improve the health status of refugees.

## Abbreviations

NHI: National Health Insurance
ANOVA: Analysis of variance
KRW: Korean won.
